# Intercellular communication in malignant pleural mesothelioma: properties of tunneling nanotubes

**DOI:** 10.3389/fphys.2014.00400

**Published:** 2014-10-31

**Authors:** Justin W. Ady, Snider Desir, Venugopal Thayanithy, Rachel I. Vogel, André L. Moreira, Robert J. Downey, Yuman Fong, Katia Manova-Todorova, Malcolm A. S. Moore, Emil Lou

**Affiliations:** ^1^Department of Surgery, Memorial Sloan-Kettering Cancer CenterNew York, NY, USA; ^2^Division of Hematology, Oncology and Transplantation, University of MinnesotaMinneapolis, MN, USA; ^3^Integrative Biology and Physiology Program, University of MinnesotaMinneapolis, Minnesota, USA; ^4^Department of Biostatistics and Bioinformatics, Masonic Cancer Center, University of MinnesotaMinneapolis, MN, USA; ^5^Department of Pathology, Memorial Sloan-Kettering Cancer CenterNew York, NY, USA; ^6^Molecular Cytology, Memorial Sloan-Kettering Cancer CenterNew York, NY, USA; ^7^Department of Cell Biology, Sloan-Kettering Institute, Memorial Sloan-Kettering Cancer CenterNew York, NY, USA

**Keywords:** tunneling nanotubes, malignant pleural mesothelioma, intercellular transfer, intercellular communication

## Abstract

Malignant pleural mesothelioma is a particularly aggressive and locally invasive malignancy with a poor prognosis despite advances in understanding of cancer cell biology and development of new therapies. At the cellular level, cultured mesothelioma cells present a mesenchymal appearance and a strong capacity for local cellular invasion. One important but underexplored area of mesothelioma cell biology is intercellular communication. Our group has previously characterized in multiple histological subtypes of mesothelioma a unique cellular protrusion known as tunneling nanotubes (TnTs). TnTs are long, actin filament-based, narrow cytoplasmic extensions that are non-adherent when cultured *in vitro* and are capable of shuttling cellular cargo between connected cells. Our prior work confirmed the presence of nanotube structures in tumors resected from patients with human mesothelioma. In our current study, we quantified the number of TnTs/cell among various mesothelioma subtypes and normal mesothelial cells using confocal microscopic techniques. We also examined changes in TnT length over time in comparison to cell proliferation. We further examined potential approaches to the *in vivo* study of TnTs in animal models of cancer. We have developed novel approaches to study TnTs in aggressive solid tumor malignancies and define fundamental characteristics of TnTs in malignant mesothelioma. There is mounting evidence that TnTs play an important role in intercellular communication in mesothelioma and thus merit further investigation of their role *in vivo*.

## Introduction

Malignant pleural mesothelioma (MPM) is a clinically devastating and locally invasive malignancy. Patients with this disease uniformly carry a poor prognosis despite advances in understanding of cancer cell biology and development of new therapies. Unlike other solid tumor malignancies, mesothelioma is highly refractory to all forms of current treatment including surgery, radiation, and chemotherapy. Treatment of mesothelioma and other invasive solid tumor malignancies such as cancers of the colon, pancreas, and ovaries is limited by an inadequate understanding of the modes and functions of intercellular communication in the tumor microenvironment (Axelrod et al., 2006; Ruckert et al., 2012). Intercellular communication is critical to tumor formation, organization, and treatment resistance (Kenny et al., 2007; Bissell and Hines, 2011; Ruckert et al., 2012). Mounting evidence suggests that tumor-stromal cell interactions are important to the invasive phenotype. Stromal cells, once seen as passive structural components of the tumor infrastructure, are now viewed as dynamic components of tumor initiation, progression, and invasion (Mueller and Fusenig, 2004; Tlsty and Coussens, 2006; Pietras and Ostman, 2010). Invasive tumors are composed of a large proportion of stroma; in MPM this proportion can be as high as 34–45% depending on the histologic subtype (Motta et al., 2006). The proportion is highest in biphasic and sarcomatoid tumors, the latter of which is associated with even worse prognosis than other subtypes (Motta et al., 2006). This tumor-stroma balance creates a heterogeneous microenvironment composed of, among other things, malignant cells, cancer-associated fibroblasts, vascular endothelial cells, macrophages, and other inflammatory infiltrates. In a study of MPM, inflammatory or desmoplastic stroma types correlated with worse patient prognosis, as compared with fibrous or myxoid forms of stroma (Cerruto et al., 2006).

The most commonly studied avenues of cellular transfer among cancer cells include gap junctions, chemokines, cytokine messengers, and microvesicles/exosomes (Bissell and Radisky, 2001; Hegmans et al., 2004; Cottin et al., 2010; Naus and Laird, 2010; Bobrie et al., 2011; Pap et al., 2011; Strassburg et al., 2012). These forms of intercellular communication are most effective over short distances. Furthermore, cell-cell junctions are disrupted upon epithelial-mesenchymal transition, a precursor to metastasis (Lamorte et al., 2002; Huber et al., 2005), making intercellular communication via these junctions impossible for separated cells. Additionally, effective cell-cell “cross-talk” via diffusible factors could be difficult to achieve because of an increase in interstitial fluid pressure in the tumor microenvironment. There remains considerable uncertainty regarding how tumor-stroma exchange of cellular information takes place and how distant cells that are not in close proximity are able to communicate within a three-dimensional matrix composed of a significant proportion of stromatous material. A better understanding of the mechanisms and cellular structures that underlie intercellular communication among distant cells in the tumor matrix of malignant tumors is expected to lead to new targeted treatments that block progression of mesothelioma and other invasive solid tumor malignancies.

Our group has investigated tunneling nanotubes (TnTs), a previously underexplored form of cellular protrusions that are distinctly unique from other filamentous cellular extensions. TnTs are long, narrow, actin-based cytoplasmic extensions that form *de novo in vitro*. Nano-sized in width (50–800 nm), TnTs can stretch the length of several cell diameters or longer (as long as several hundred microns) to form direct cell-to-cell cytoplasmic connections. TnTs display non-adherence to the substratum when cultivated *in vitro* (Rustom et al., 2004). These characteristics differentiate TnTs from other, well-known actin-based cytoplasmic extensions including lamellopodia, filopodia, and invadopodia (Rustom et al., 2004). TnTs are open-ended “intercellular bridges” whose walls consist of a contiguous lipid bilayer that can establish a direct connection between the cytoplasm of connected cells, or in some cases interface with gap junctions in plasma membranes (Wang et al., 2010). TnT formation is largely generated by actin-driven membranous protrusions extending to outlying cells. They have been noted to form either by one cell extending a tubular cytoplasmic connection to another cell located at some distance (in contrast with gap junctions, which connect cells in immediate proximity) or to form between cells in close proximity that then move apart via usual mechanisms of cell motility, allowing for continuation of intercellular communication even as the cells move in different directions (Veranic et al., 2008). At least one study has suggested that TnTs interface with gap junctions to connect cells and mediate intercellular cross-talk (Wang et al., 2010). Uniquely, TnTs serve as conduits for intercellular shuttling of cellular organelles and other cargo between connected, non-adjacent cells (Lou et al., 2012a,b). *In vitro* studies have shown that TnTs have the ability to directly mediate cell-to-cell communication by serving as long-range conduits between connected cells for intercellular transfer of proteins, mitochondria, Golgi vesicles, and even viruses (Koyanagi et al., 2005; Onfelt et al., 2005, 2006; Sherer et al., 2007; Davis and Sowinski, 2008; Sherer and Mothes, 2008; Plotnikov et al., 2010; Yasuda et al., 2010; He et al., 2011; Kadiu and Gendelman, 2011; Wang et al., 2011; Lou et al., 2012b) (For an example of time-lapse imaging we use in our work, please see Movie S1 demonstrating intercellular transfer of mitochondria between mesothelioma cells connected via nanotube). The importance of intercellular transfer of genetic material is also a topic of growing interest. Our group recently demonstrated that TnTs can also transport oncogenic microRNAs between malignant cells, as well as between malignant and stromal cells, introducing a new aspect of tumor-stromal cross-talk that warrants further study (Thayanithy et al., 2014a).

TnTs have been studied in a wide variety of non-cancer cell types including dendritic cells and monocytes (Watkins and Salter, 2005; Salter and Watkins, 2006), mature macrophages (Eugenin et al., 2009; Hase et al., 2009), T cells (Sowinski et al., 2008, 2011; Rudnicka et al., 2009), B cells (Xu et al., 2009), neutrophils (Galkina et al., 2010), neuronal cells (Gousset et al., 2009), kidney cells (Gurke et al., 2008), endothelial progenitor cells (Yasuda et al., 2010), mesothelial cells (Ranzinger et al., 2011; Lou et al., 2012b), cardiomyocytes (Koyanagi et al., 2005), and mesenchymal stromal cells (Cselenyak et al., 2010; Plotnikov et al., 2010). Our group focuses on investigation of TnTs in the context of invasive forms of cancer (Lou et al., 2012a,b). To investigate TnTs as a physiologically relevant structure in human solid tumor malignancies, our initial work successfully visualized TnTs in solid tumors resected from patients with mesothelioma and lung adenocarcinomas (Lou et al., 2012b), providing the first evidence of the potential *in vivo* relevance of these cellular structures in cancer. We subsequently performed high-resolution microscopy and 3-dimensional reconstructions to confirm that nanotube structures are present in other invasive malignancies as well, including a murine model of osteosarcoma and human ovarian adenocarcinoma (Thayanithy et al., 2014a). In our *in vitro* work in mesothelioma, we used modified wound-healing assays and demonstrated TnT formation along the leading invasive edge of mesothelioma cells *in vitro*; time-lapse imaging revealed regular formation of TnTs by proliferating and migrating mesothelioma cells advancing to fill the gap (Lou et al., 2012b). This finding introduces the possibility that TnTs facilitate intercellular communication and the progression of malignancy at the leading edge of invasive mesothelioma tumors. More recently, we showed that exosomes and TnTs may work synergistically by demonstrating that exogenous tumor exosomes induced an increased rate of TnT formation (Thayanithy et al., 2014b). Electron microscopy revealed that exosomes were located at the base of TnTs and in the extracellular environment. Our subsequent studies identified enrichment of lipid rafts, small intra-cytoplasmic cholesterol microdomains, in mesothelioma cells connected via nanotubes (Thayanithy et al., 2014b). These findings implicate exosomes as potential chemotactic stimuli for TnT formation and lipid rafts as a potential biomarker for TnTs. The effects of TnT formation and TnT-mediated transport of cellular cargo on malignant cell behavior are currently unknown.

In the current study, we sought to further characterize the properties of TnTs in mesothelioma, including differences in formation of TnTs between malignant mesothelioma cells and non-malignant mesothelial cells; quantitative differences in TnT length in relation to cell proliferation; properties of TnT formation in clinically relevant models, such as between non-adherent cells, mimicking the scenario of mesothelioma cells floating in peritoneal or thoracic effusions as a hallmark of malignant progression; and structural components of TnTs in mesothelioma cells. Finally, we also sought to develop new approaches to 3-dimensional *in vitro* and *in vivo* modeling for the study of TnTs in tumor propagation and resistance to therapy.

## Materials and methods

### Cell lines and culture media

MSTO-211H cells were derived from a patient with biphasic mesothelioma (ATCC no. CRL-2081); VAMT is a sarcomatoid mesothelioma cell line; and H2052 is a mesothelioma cell line of epithelioid histology. All three mesothelioma cell lines (MSTO-211H, VAMT, and H2052) were passaged using 10% fetal bovine serum (FBS) in RPMI-1640 with 25 mM glucose, supplemented with 1% penicillin-streptomycin (P-S) and 2% L-glutamine, at normal pH (7.6). The normal immortalized human mesothelium cell line MeT5A was passaged in 10% FBS in M199/MCDB105 (1:1) with 100 U/ml penicillin-streptomycin and 2% L-glutamine. All cell lines used in this study were authenticated by the Core Fragment Analysis Facility at Johns Hopkins University using short tandem repeat profiling. Cells were passaged in 75 cm^2^ tissue culture flasks (Falcon, Becton Dickson, Oxnard, CA) at 37°C in 5% CO_2_. Nanotube formation was stimulated by growing cells in 2.5% FBS in RPMI-1640 containing 50 mM glucose, supplemented with 1% P-S and 2% L-glutamine as described previously (Lou et al., 2012b); we refer to this combination throughout the text as “TnT medium.” For 3-dimensional *in vitro* modeling, we mixed MSTO-211H cells in 5% FBS in RPMI medium containing 100 mM glucose in a 1:1 ratio with 2% agarose to compose a final medium composed of 1% agarose and TnT medium for further culture of cells. These cells were then added to 6-well culture plates for microscopic examination.

### Quantification of TnTs per cell

Three MPM cell lines (H2052, VAMT, and MSTO-211H) and one benign mesothelial cell line (MeT5A) were plated at a density of 6 × 10^4^ cells/well in 6-well adherent tissue culture plates (Fisher Scientific, Pittsburgh, PA) at 37°C in 5% CO_2_ with TnT-inducing medium (described above). TnTs were identified using the parameters described by Rustom et al. (2004) as well as in our previous publications (Lou et al., 2012b; Thayanithy et al., 2014b). Briefly, these parameters included (i) lack of adherence to the substratum of tissue culture plates, including visualization of TnTs passing over adherent cells; (ii) TnTs connecting two cells or extending from one cell were counted if the width of the extension was appropriately narrow and estimated to be <1000 nm in width; and (iii) a narrow base at the site of extrusion from the plasma membrane. Cellular extensions not clearly consistent with the above parameters were not included in the final count. An Olympus IX70 inverted microscope (Olympus Corporation) with 20× objective lens was used to count the number of TnTs and cells in 10 randomly chosen fields of each 6-well plate at 24, 48, and 72 h. A single representative image was taken at all time points for each well for analysis of TnT length. Experiments were performed in duplicate for each cell line. The number of TnTs per cell (TnTs/cell) was counted to exclude the possibility that increases in TnTs were due to increased cell proliferation. Means were calculated and compared using two-sided, two-tailed *t*-tests assuming unequal variances. *P*-values <0.05 were considered statistically significant.

### Quantification of TnT length

Representative images taken from the previous experiment were analyzed using ImageJ software. TnT length was measured by normalizing the 200 micron scale bar from the images to the number of pixels. The length of TnTs from each cancer cell line was measured at 24, 48, and 72 h. TnT lengths were not normally distributed; therefore, Wilcoxon Rank Sum tests were used to compare TnT lengths for each combination of time measurements within each cell line. *P*-values <0.05 were considered statistically significant.

### TnT tethering assays

Pleural effusion or ascites specimens from cancer patients with MPM and lung adenocarcinoma were obtained via a Memorial Sloan-Kettering Cancer Center (MSKCC) Institutional Review Board (IRB)-approved protocol. Informed written consent was obtained from all patients, and patient identifiers were removed to ensure anonymity. Malignant cells were histologically confirmed by an experienced MSKCC pathologist and seeded in standard tissue culture-treated plates using a clonal dilution assay. Cells were seeded in 24-well non-adherent culture (Nunc Non-Treated Multidishes) and adherent treated tissue culture plates (Fisher Scientific, Pittsburgh, PA) using 10% FBS RPMI. Microscopic imaging was used to confirm the presence of single cells per well, and these wells were marked and monitored daily by microscopic imaging. We additionally performed similar experiments with mesothelioma cell lines VAMT, H2052, and MSTO-211H using an identical approach.

### Fixation and sample preparation

To prepare cells for IF staining, cells were cultured in one- or two-well sterile tissue culture-treated chamber slides (Lab-Tek II Chamber Slide™ system, Nunc, Rochester, NY) or on sterile poly-L-lysine (1 mg/ml; Sigma) coated glass coverslips (VWR VistaVision, catalog no. 16004-312) for 48–72 h using TnT medium to stimulate TnT formation. As TnTs are highly sensitive to movement and to light, we have modified existing protocols for cell fixation and analysis. To perform fixation and prevent disruption of existing nanotubes, 16% w/v paraformaldehyde (PFA) (Alfa Aesar, Ward Hill, MA) was added along the sides of the chambers or the dishes with glass coverslips, keeping the overlying culture medium intact to a final w/v concentration of 4%, After incubation at 4°C for 1–2 h, the fixative and chambers were removed, and slides were allowed to air dry. We have found this combination provides optimal preservation of intact cells with TnTs to allow for more accurate study of these thin structures. Immunofluorescent staining was then performed to detect expression of the noted proteins.

### Immunofluorescent staining

The primary antibodies and their working concentrations are as follows: cdc42 (Santa Cruz Biotech, 200 ug/ml, rabbit polyclonal IgG; catalog no. SC-87); NF2/merlin (Santa Cruz Biotech, 200 ug/ml, rabbit polyclonal IgG; catalog no. SC-332), p-selectin (CD62P) (BD Biosciences, 5 μg/ml; catalog no. 556087), beta-tubulin (Sigma–Aldrich, monoclonal anti-acetylated tubulin, clone 6-11B-1; catalog no. T6793-0.2ML); monoclonal anti-β-Tubulin IV (Sigma–Aldrich, catalog no. T7941, clone ONS.1A6); vimentin Alexa Fluor 488 (BD Pharmingen, human IgG; catalog no. 562338), Akt (Sigma–Aldrich, rabbit polyclonal IgG; catalog no. AAB4300259-100UG). Slides were first blocked with blocking solution (10% normal goat serum/2% BSA in PBS) or mouse IgG blocking agent from Vector Labs (catalog no. MKB-2213) for 30 min. Primary antibody incubation lasted 3–7 h at room temperature, followed by 30 min incubation with biotinylated secondary antibodies (Vector Labs, MOM Kit BMK-2202; 1:200 dilution). Detection was performed with Streptavidin-HRP D (Ventana Medical Systems) followed by Tyramide-Alexa Fluor 488 (Invitrogen, catalog no. T20922).

### Drug treatment of cells with migrastatin

Migrastatin core ether is a synthetic analog of migrastatin obtained courtesy of Dr. Samuel Danishefsky; it was used at 100 nM. MSTO-211H cells were prepared as above (i.e., 1 × 10^5^ cells per well in 24-well tissue culture plates). The number of TnTs was counted in 10 fields per medium condition, at regular time intervals (0, 24, 48, and 72 h) using a 20× objective lens on a Nikon Eclipse Ti inverted microscope (Nikon Instruments, Inc.). Experiments were performed in duplicate. Means were calculated and compared using two-sided, two-tailed *t*-tests assuming unequal variances. *P*-values <0.05 were considered statistically significant.

### Treatment of cells with other drugs (tunicamycin, dextran sulfate, 4-methylumbelliferone) to assess potential association of hyaluronan with TnTs

Tunicamycin (Sigma, catalog no. T7765-1MG, lot no. CAS 11089-659) was used at a final concentration of 5 μg/ml; Poly I:C (Polyinosinic–polycytidylic acid sodium salt; Sigma, catalog no. P0913-10MG, lot no. CAS 42424-50-0) was used at a final concentration of 10 μg/ml; dextran sulfate (Sigma, catalog no. D8906-5G) stock solution was made by first dissolving in 2% FBS in phosphate-buffered saline (PBS) to create a stock solution of 100 μg/ml, which was then added to culture medium to final concentration 10 μg/ml; 4-methylumbelliferone (4-MU; Sigma, catalog no. M 1381) was prepared as a stock solution 20 mg/ml concentration in DMSO, then added to culture medium to final concentration 1.0 mM; Hyaluronidase (Sigma, H3884) was prepared as a stock solution of 10 mg/ml and used at a final concentration of 13 μM. For preparation of each drug, stock solution was added to 10% FBS RPMI-1640 medium to obtain final concentrations as listed; this medium was used in cell culture by adding to 1 × 10^4^ MSTO-211H cells per well of 6-well tissue-culture treated plates (Fisher Scientific, Pittsburgh, PA). Following 48 h of incubation at 37°C (5% CO_2_), exclusion assays were performing by adding either U937 mononuclear cells at 4°C for 1 h or red blood cells (erythrocytes) as noted in the Results Section and per standard protocols (DiCorleto and de la Motte, 1985; Rilla et al., 2008), followed by microscopic imaging. For fluorescent imaging, MSTO-211H cells transfected with a lentivirus expressing green fluorescent protein (GFP) were used along with U937 cells transfected with a lentivirus expressing Tomato Red.

### *In vivo* growth of mesothelioma cells preconditioned with TnT medium

GFP-luciferase expressing MSTO cells were grown in either of two conditions: normal RPMI (10% FBS RPMI, 25 mM glucose) or TnT-inducing medium, which consists of low serum and high glucose RPMI (2.5% FBS RPMI, 50 mM glucose), for 7 days. Both sets of media were supplemented with 1% penicillin/streptoymycin and 2% L-glutamine. All cells were cultured in 6-well adherent tissue culture plates (Fisher Scientific, Pittsburgh, PA) at 37°C in 5% CO_2_. Cells (2.9 × 10^5^) were then suspended in 100 μL of RPMI and injected into the peritoneum of each mouse. Ten NOG (NOD/Shi-scid/IL-2Rˠ^null^) immunodeficient mice were used for each group. Each mouse was concurrently injected intraperitoneally with 1 ml of thioglycolate as a co-stimulatory inflammatory agent; the rationale for this is that inflammation is known to elicit formation of nanotubes in *in vivo* animal models (Seyed-Razavi et al., 2013). On days 7, 14, 21, and 31 post-tumor inoculation, mice were anesthetized with isoflurane and injected intraperitoneally with 150 μ L of luciferin (15 mg/mL). Mice were then imaged with an Ivis 200 optical imaging system (Caliper Life Sciences, Hopkinton, MA) 5 min after injection. Capture time was 40 s. Living Image software version 2.5 was used to quantify average radiance (p/s/cm2/sr). Means were calculated and compared using two-sided, two-tailed *t*-tests assuming unequal variances. Overall survival (OS) of the mice was calculated from date of tumor inoculation to date of death, or censored at 40 days for those still alive at the end of the experiment. OS was summarized using a Kaplan-Meier curve and a comparison between groups was made using a Log-Rank test. *P*-values <0.05 were considered statistically significant.

### Sectioning, staining, and imaging human tumor samples

Tumor specimens from patients with MPM were obtained via a MSKCC IRB-approved protocol. Informed written consent was obtained from all patients, and patient identifiers were removed to ensure anonymity. Resected intact tumor specimens were placed in PBS. Vibratome sections (100–300 mm thick) were cut and stained using Hoechst 33342 (10 mg/ml) and MitoTracker Red dyes (500 nM) using protocols we developed and have described previously (Lou et al., 2012b). Stained sections were mounted between two glass coverslips and imaged on a confocal microscope.

### Optical imaging of human tumor samples and image processing

Confocal imaging of samples was performed using Zeiss LSM 5Live line-scanning or Leica SP2 point-scanning microscopes using Zeiss oil 406/1.3NA Plan-Neoflur, Zeiss oil 636/1.4NA Plan-Apochromat or Leica water 636/1.2NA HCX PL APO CS objectives. Serial z-stack images were obtained at optimal step size and maximum intensity projection images were produced. The Imaris Viewer program (Bitplane Scientific Software, Inc.) was used to construct and visualize 3-dimensional images of tumor samples. Metamorph (Molecular Devices) image analysis software was used to create still images and movies.

### Electron-microscopic imaging of nanotubes

To perform scanning and transmission EM, 1–3 × 10^6^ MSTO-211H cells were cultured on Thermanox plastic tissue culture 25 mm cover slips (Lux Scientific Corporation). The fixative—2.5% glutaraldehyde/2% paraformaldehyde in 0.075 M sodium cacodylate buffer (pH 7.5; 10 ml, Electron Microscopy Sciences, Hatfield, PA)—was added directly to the overlying medium.

### Cell culture and RNA isolation

MSTO-211H cells (8 × 10^5^) were seeded in 150 cm^2^ flasks and grown for 7 days using passage medium or TnT medium at 37°C in a standard humidified chamber with 5% CO2 as already described in the text. After 7 days, the cells were harvested separately following trypsinization and subjected to RNA isolation using mirVana™ total RNA isolation protocol following the protocol of the manufacturer (Life Technologies, Carlsbad, CA). RNA preparation was quantified on a Nanodrop spectrophotometer (Thermo Fisher, Wilmington, DE, USA), and RNA quality was confirmed by resolving on a denaturing 1.2% agarose gel following standard electrophoresis protocol.

### cDNA synthesis and qRT-PCR

RNA was subjected to first strand cDNA using miScript II Reverse Transcription kit (Qiagen, Valencia, CA). Total RNA was reverse transcribed for 2 h at 37°C, and reverse transcriptase was heat inactivated by boiling the reaction mix at 95°C for 5 min. cDNA (5.0 ng) was diluted and amplified with 10 μl of miScript SYBR green PCR mix following the protocol of the manufacturer using gene-specific forward and reverse primers. PCR primers were purchased from a commercial vendor (IDT, Coralville, IA). The nucleotide sequence of the primers used are listed in Supplementary Table 1. The samples were run in triplicate in a Roche Light Cycler II (Roche GmbH, Germany), and values were normalized to the endogenous expression of 18S rRNA. Fold gene expression was calculated by comparative C(T) method (Schmittgen and Livak, 2008) and mean fold expression values relative to expression in control medium were compared using two-sided two-sample *t*-tests assuming unequal variances. *P*-values <0.05 were considered statistically significant.

### Time-lapse imaging of cells in culture

Time-lapse imaging experiments were performed on Perkin Elmer UltraView ERS spinning-disk confocal microscope or Zeiss LSM 5Live line-scanning confocal microscope. Both microscopes were enclosed in environmental chambers that were maintained at 37°C with 5% CO_2_ level. Viable Staining of Cell Lines for time-lapse imaging was performed as we have described previously (Lou et al., 2012b). Briefly, in order to assess the ability of mitochondria to be transmitted between mesothelioma cells via TnTs, we used MitoTracker Red to stain MSTO-211H cells which were then cultured in hyperglycemic, low-serum (“TnT”) medium. The cells were cultured in clear-bottomed delta-T culture dishes (Bioptechs Inc., Butler, PA). MitoTracker Red CMX Ros (Invitrogen, M-7512, 50 μg/vial) was used at 500 nM to stain mitochondria, per manufacturer's protocols. Stained cells were re-suspended and added to a non-confluent culture of adherent, unstained MSTO-211H cells grown in another dish. Incubation was performed in high glucose medium for 5 h to stimulate formation of TnTs prior to imaging.

## Results

### Malignant mesothelioma cells form more TnTs than benign mesothelial cells

**Figure 1:**
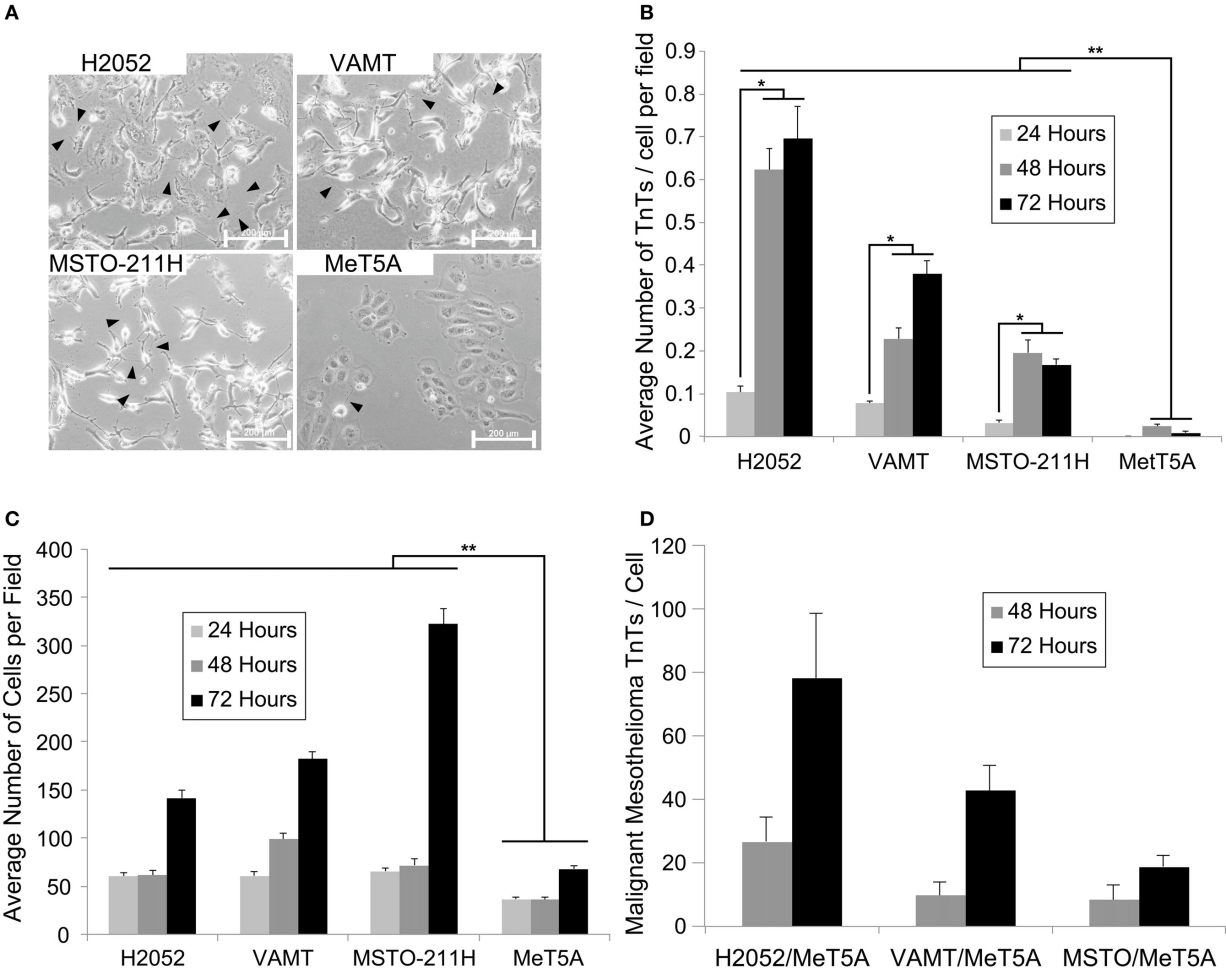
**Malignant mesothelioma cells form more TnTs than benign mesothelial cells**. An Olympus IX70 inverted microscope with 20× objective lens was used to count the number of TnTs and cells in 10 randomly chosen fields at 24, 48, and 72 h. Representative images of TnTs of three malignant mesothelioma cell lines (H2052, VAMT, MSTO-211h) and a benign mesothelial cell line (MeT5A) were evaluated and are shown **(A)**. Arrowheads indicate nanotubes. The number of TnTs per cell (TnTs/cell) was counted **(B)** to exclude the possibility that increases in TnTs were due to increased cell proliferation **(C)**. Experiments were performed in duplicate for each cell line and results were averaged. Means ± standard errors are plotted. Double asterisks indicate statistically significant differences (*p* < 0.05) between each mesothelioma cell line compared to the mesothelial cell line; single asterisks indicate statistically significant differences between timepoints within each malignant mesothelioma cell line. Also shown is a ratio of TnTs per cell at 48 and 72 hours comparing individual malignant cell lines to MeT5A **(D)**.

We propose that TnTs create intercellular networks with the capability of transmitting signals that stimulate proliferation of invasive cancers. To determine whether TnT formation occurs at a higher rate in malignant mesothelioma cells than in benign cells, we cultured the MPM cell lines H2052, VAMT, and MSTO-211H and the benign mesothelial cell line MeT5A. Cells were cultured in medium that we previously demonstrated induces TnT formation (Lou et al., 2012b). Equivalent numbers of cells were added to culture wells and visualized using inverted microscopy at 24, 48, and 72 h; representative images are shown in the accompanying figures (Figure 1A; also Supplemental Figure S1 for composite panel of representative images at 24, 48, and 72 h). At each time point, we randomly selected 10 fields of view using the 20× objective and counted the number of TnTs per field. We also counted the number of cells present in each selected field to control for cellular proliferation. For all three malignant mesothelioma cell lines, the average number of TnTs/cell was significantly higher than that seen for the benign mesothelial MeT5A cell line (Figure 1B). No evidence of TnT formation was evident in the mesothelial cell line (MeT5A) at 24 h, and thus a ratio could not be reported. As expected, cell proliferation was higher in malignant mesothelioma cell lines as compared to normal MeT5A cells (Figure 1C); however, among malignant cells proliferation appeared to be inversely associated with the rate of TnT formation. The ratio of malignant:mesothelial TnTs/cell doubled or tripled from 48 to 72 h for all malignant cell lines (26.73–78.16 for H2052, 9.80–42.66 for VAMT, and 9.80–18.84 for MSTO) (Figure 1D). Taken together, these *in vitro* data show that TnTs formed at a markedly higher rate among malignant mesothelioma cell lines than among normal mesothelial cells in inverse proportion to the rate of cell proliferation, ranging from nearly 20-fold to 80-fold higher by 72 h of *in vitro* culture. Moreover, these data support the use of a “nanotube index” to quantitatively assess TnT formation in future studies of the biological role of TnTs in aggressive malignancies. This markedly higher rate of TnT formation in mesothelioma, and likely in other cancers as well, provides evidence to support TnTs as a potential novel target for selective therapy of such cancers.

### Overall TnT length decreases with time and with proliferation of mesothelioma cells

**Figure 2:**
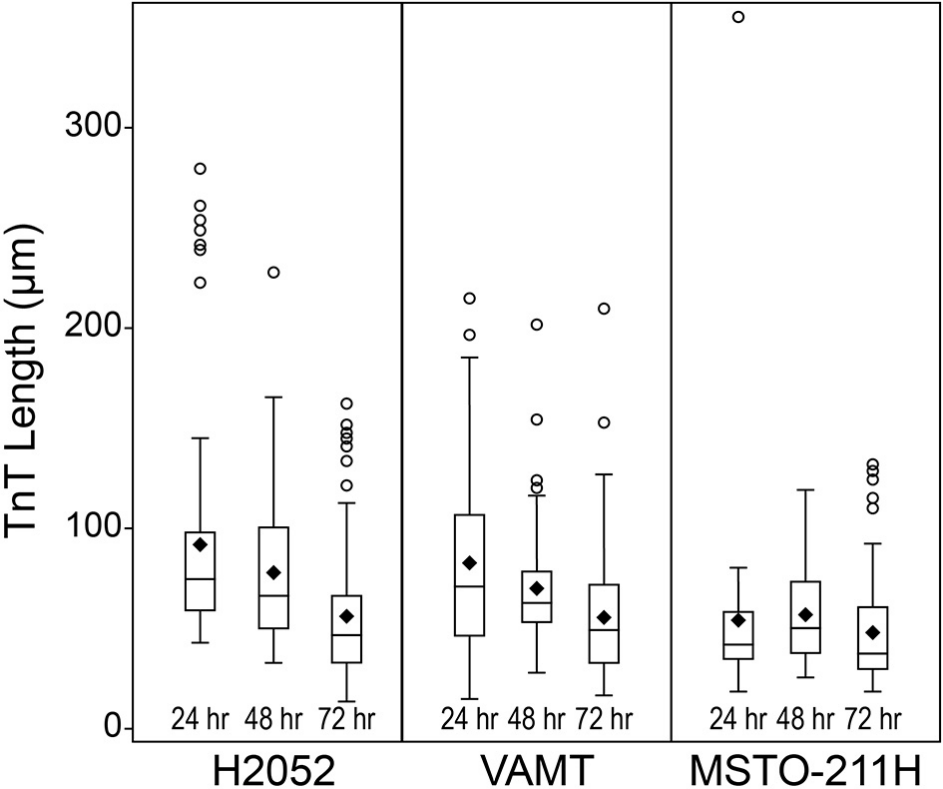
**TnT length decreases with time and with proliferation of mesothelioma cells**. Microscopy images were taken of cells forming TnTs at 24, 48, and 72 h. Images were representative of the 10 randomly chosen fields from Figure 1. Average TnT length was estimated through measurement of image pixels. Box plot depicts distribution of lengths of TnTs in the malignant mesothelioma cell lines. Symbols on the boxplot are as follows: Box, 1st to 3rd (Q1–Q3) Quartiles; Diamond, Mean; Line inside box, Median; Circle, Outlier.

In the context of a heterogeneous tumor matrix, TnTs may play an important role in long-distance cellular communication. To accomplish this, TnTs would need to extend to variable lengths depending on the distance of targeted cells. As more cells accumulate, this distance would become shorter. We hypothesized that TnT length would decrease as cells proliferate and accumulate over time in *in vitro* culture. We cultured the MPM cell lines H2052, VAMT, and MSTO-211H and the mesothelial cell line MeT5A. TnT length decreased over time among all malignant cell lines (Figure 2); these changes were statistically significant at most time points (Table 1). TnT length decreased slightly from day 2 to day 3 among the non-malignant MeT5A cells (Figure 2); however, this change was not statistically significant. We depict the data in the form of box plots in order to demonstrate the median and the wide range of lengths we observed in the malignant mesothelioma cell lines (Figure 2). Since TnT formation between MeT5A cells was rare, it was not possible to construct box plots for the distribution of TnT length over the three-day period for this non-malignant cell line. The decrease in TnT length observed among malignant cells was most noticeable for H2052 and VAMT cells, but was less dramatic for MSTO cells. This difference could be due to the relatively steady rate of proliferation of H2052 and VAMT cells and high proliferation rate of MSTO cells (Figure 1C). In addition, this finding is consistent with our prior study showing that TnTs are most prominent in sub-confluent cultures; in fully confluent cultures, cells are in close proximity, making it either difficult to discern any present TnTs and/or decreasing the number of TnTs that form in conditions that do not require long-distance connections (Lou et al., 2012b).

**Table 1:** ***P*-values from comparisons of TnTs Length in mesothelioma cells by cell line and time**.

**Cell lines:**		**H2052**	**VAMT**	**MSTO-211H**
Time (hours)	24 vs. 48	*P* = 0.112	*P* = 0.314	*P* = 0.075
	24 vs. 72	*P* < 0.0001	*P* < 0.0001	*P* = 0.386
	48 vs. 72	*P* < 0.0001	*P* < 0.0001	*P* = 0.002

*P-value ≤ 0.05 was considered significant*.

### TnT tethering of mesothelioma cells in *in vitro* model of pleural effusions

**Figure 3:**
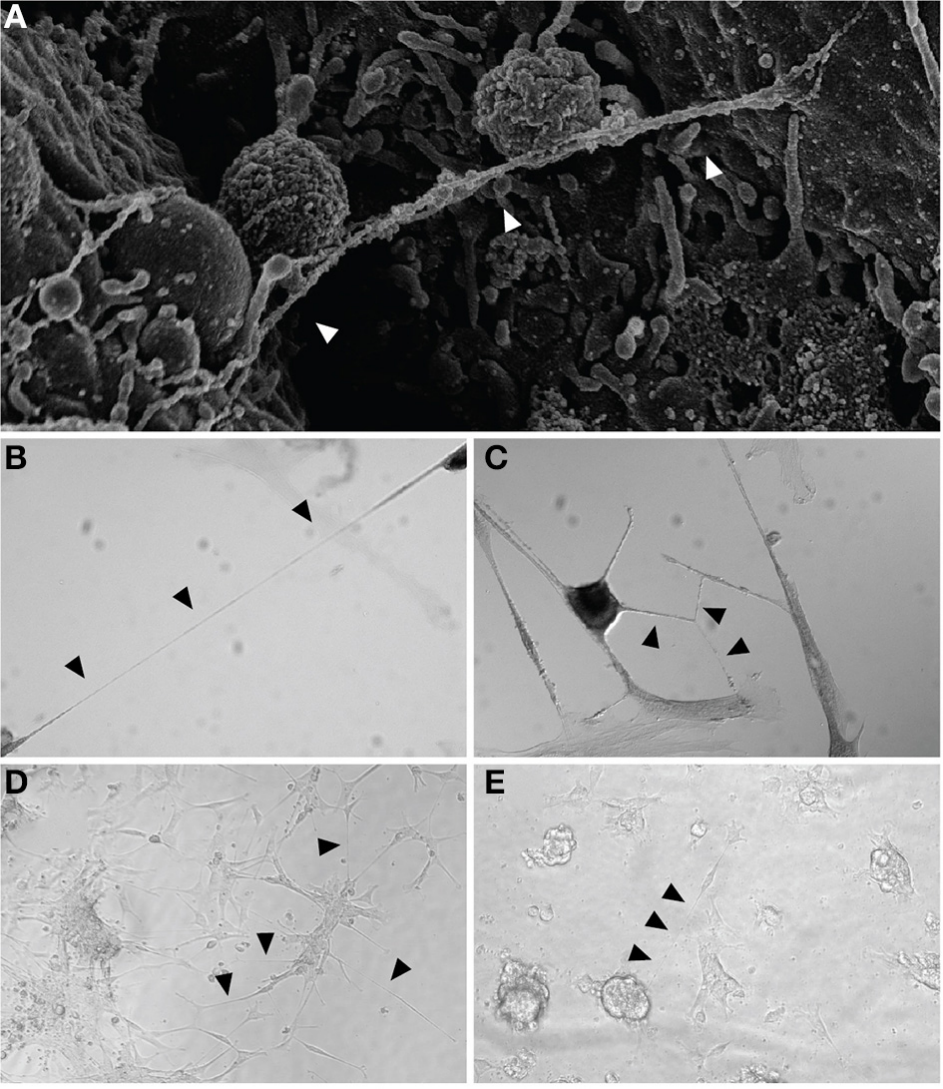
**TnT tethering of mesothelioma cells in *in vitro* models of pleural effusions. (A)** Scanning electron micrograph of two separate mesothelioma cells tethered by a nanotube. **(B,C)** TnTs connecting primary malignant cells from pleural effusions. 40× magnification. **(D,E)** Clonal dilution assay using VAMT (sarcomatoid mesothelioma) cells grown in a low-adherence culture plate using 10% FCS RPMI medium; cells are shown on day 24 of culture in separate wells from the same plate. Arrowheads indicate nanotubes.

Advanced thoracic malignancy is frequently associated with accumulation of malignant fluid in the pleural or peritoneal cavities. These effusions often contain detached, free-floating suspended malignant cells that are capable of undergoing epithelial-to-mesenchymal transition (EMT), thus increasing their invasive capability in some cancer types, such as lung adenocarcinoma (Chen et al., 2013; Chunhacha et al., 2013). In our prior study, we demonstrated that EMT effectively stimulates TnT formation in mesothelioma (Lou et al., 2012b). We hypothesized that non-adherent viable cells in culture in essence behave similarly to suspended effusion cells in mesothelioma patients. We thus next obtained pleural effusion specimens from 5 patients diagnosed with MPM or lung adenocarcinoma. After isolating malignant cells via centrifugation of pleural effusions, we confirmed the presence of malignant cells by morphology and TnT formation among these cells by inverted as well as by scanning electron microscopy (Figure 3A). We then cultured cells *in vitro* in standard tissue culture-treated plates and confirmed formation of TnTs connecting these patient-derived, primary malignant cells (Figures 3B,C).

We noted that malignant cells presented in effusions as dispersed single cells or as spheroid aggregates that could be disassembled into single cells through trypsinization or by physical separation with vigorous pipetting. We hypothesized that clusters of cells derived from a single parent cell could form TnTs in suspension and without full cell adherence to the substratum of the culture plate. To investigate this possibility, we performed separate clonal dilution assays using VAMT and H2052 cell lines cultured in 24-well non-adherent culture plates. We visually confirmed the presence of single cells and marked these wells for further daily follow-up. We performed daily microscopic imaging and reproducibly detected growth of groups of cells derived from parent cells forming mesh-like syncytial networks of TnTs connecting daughter cells. Cell aggregates formed prominently under non-adherent culture conditions while maintaining extensive TnT connections; these aggregates were in many instances connected to each other by nanotube structures while remaining suspended in culture medium (Figures 3D,E; also Supplemental Figure S2). As TnTs are in essence 3-dimensional structures (i.e., non-adherent to the substratum), they may “oscillate” upon movement of the culture plate. Other actin-based structures, such as invadopodia, do not demonstrate this trait. Using fine focus to evaluate TnTs in live cell culture also helps to differentiate TnTs from other cell extensions. The observation that primary malignant cells formed a syncytial “network” connected by TnTs indicates that these or other cytoplasmic extensions may play an unrecognized role in facilitating communication among suspended malignant cells and malignant/mesothelial cells adherent to the pleural lining of the thoracic cavity.

These data suggest that TnTs may play a role in tethering suspended, non-adherent cells. As development of pleural effusions or abdominal ascites is a hallmark of a number of aggressive solid tumor malignancies—most especially malignant pleural and peritoneal mesotheliomas, as well as lung adenocarcinomas—these results provide potential insight into the cellular behavior of malignant cells at the advanced stages of cellular invasion. They also build upon our work demonstrating TnT formation between primary malignant mesothelioma cells *in vitro* (Lou et al., 2012b) as well as similar work demonstrating TnTs between human peritoneal mesothelial cells in culture (Ranzinger et al., 2011). Further *in vivo* studies will be necessary to clarify whether such TnT connections occur among malignant pleural or peritoneal mesothelioma cells invading the mesothelial lining.

### Drug inhibition of mesothelioma TnTs using migrastatin, an inhibitor of fascin

**Figure 4:**
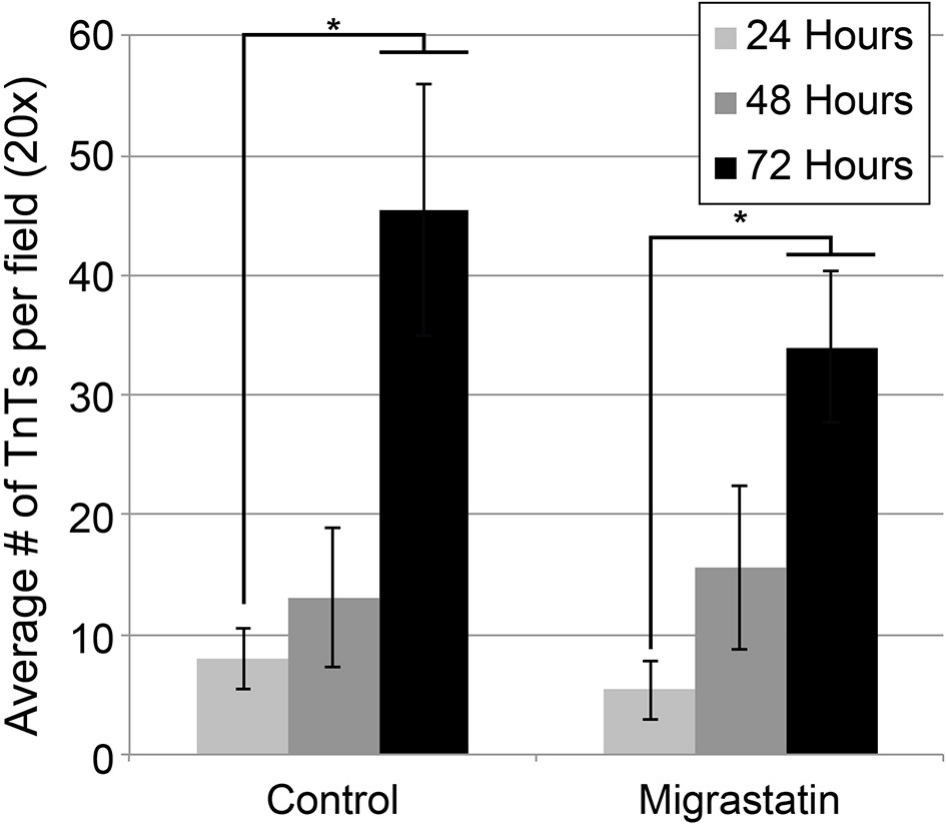
**Effect of Migrastatin on TnT formation in MSTO cells**. Migrastatin was used at 100 nM. TnTs were counted in 10 fields per timepoint per condition using 20× objective lens, and the results averaged. Experiments were performed in duplicate. Comparisons between Migrastatin and the Control were statistically significant at 24 h (*p* = 0.036) and at 72 h (*p* = 0.010). Means ± standard deviations are presented. Asterisk indicates statistical significance (*p* < 0.05).

We previously demonstrated that fascin localizes at the site of TnT extrusion from the plasma membrane at the leading edges of cells (Lou et al., 2012b). To determine whether fascin inhibition blocks TnT formation, we used migrastatin core ether (ME), a drug derived from migrastatin, a polyketide product initially derived from *Streptomyces* (Oskarsson et al., 2010). Synthetic analogs of migrastatin inhibit migration of cancer cells (Oskarsson et al., 2010) by targeting fascin and thereby blocking tumor progression (Chen et al., 2010). MSTO cells treated with ME exhibited a statistically significant difference with fewer TnTs, compared with the Control group without drug treatment, at 24 h (*p* = 0.036) and at 72 h (*p* = 0.010) (Figure 4). There was not significant difference at the 48-h timepoint.

### Three-dimensional *in vitro* model of mesothelioma tumor microenvironment

**Figure 5:**
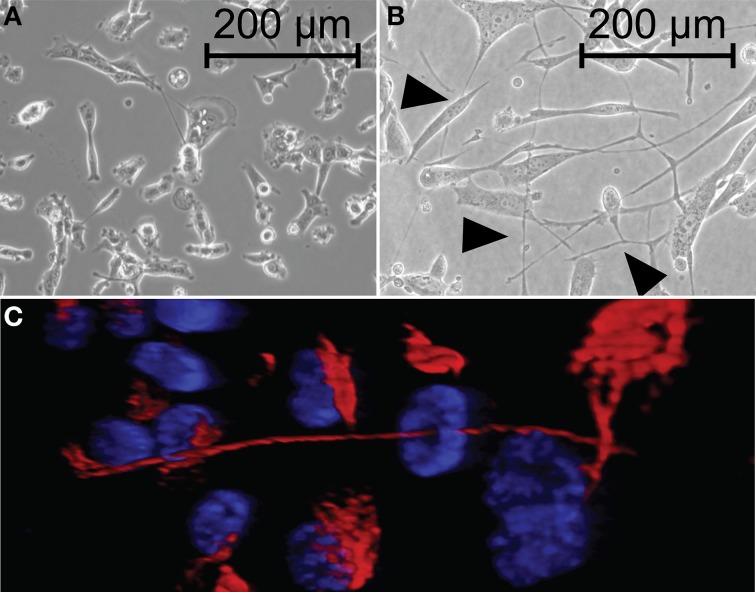
**3-dimensional modeling of TnTs *in vitro*. (A)** MSTO cells forming TnTs in regular tissue culture treated plate; **(B)** MSTO cells cultured in 1% agarose, forming TnTs vertically as well as horizontally within the agarose matrix. **(C)** 3-dimensional modeling of TnT connecting cells using confocal imaging with z-stacking (IF staining performed using fluorescent vimentin-specific antibody); also see Movie S2. Arrowheads indicate nanotubes.

Routine use of 3-dimensional modeling both *in vitro* and eventually *in vivo* will be critical to advancing the field of TnT biology, in cancer and in other diseases. To develop a 3-dimensional *in vitro* model of the tumor microenvironment, we used 1% agarose to culture mesothelioma cells to simulate suspension of cells within a 3-D viscous matrix. TnTs visualized in the 3D matrix were readily seen forming TnTs vertically and horizontally within the agarose matrix as compared to 2D tissue culture (standard tissue culture in Figure 5A; with TnT medium in 1% agarose, Figure 5B). Additionally, z-stacked confocal imaging of TnTs connecting cells stained with immunofluorescent antibodies can be used to visualize TnTs 3-dimensionally. Using this technology, we depicted a representative TnT stained with an immunofluorescent antibody to vimentin (Figure 5C; Movie S2, depicting rotating 3-dimensional model of this image). Immunofluorescence staining indicated the presence of vimentin along the length of TnTs.

**Figure 6:**
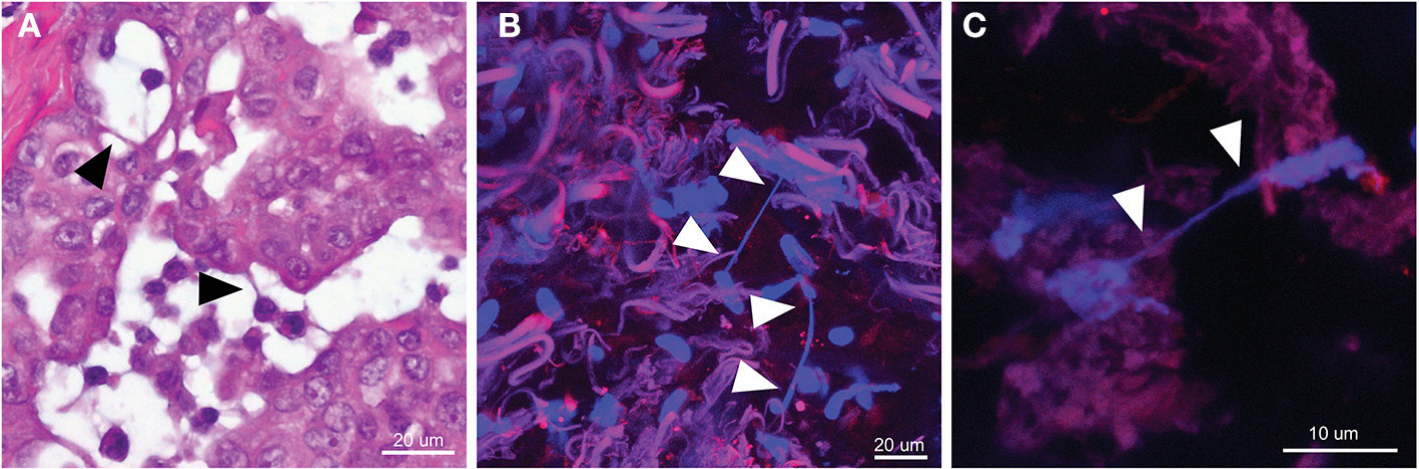
**Comparison of 2-dimensional histopathology section vs. 3-dimensional confocal imaging of primary human malignant mesothelioma tumors *ex vivo*, with specific examination for TnTs. (A)** Hematoxylin and eosin (H&E) staining of sectioned primary human mesothelioma tumor; arrowheads indicate putative nanotube-like structures; **(B,C)** compiled confocal image produced by combining individual z-stacked images of representative portions of tumor tissue resected from a human patient. Tumors were stained with MitoTracker and Hoechst 334 dye; arrowheads indicate nanotube structures. Please also see Movie S5.

In the case of solid tumor malignancies, including mesothelioma, standard and conventional evaluation of tumors involves histopathologic analysis of extremely thin 2-dimensional tumor sections (Figure 6A). While slides prepared in this manner may potentially yield views of putative nanotube-like structures, advances in microscopic imaging are required to more effectively study 3-dimensional tumors. Advances in microscopic imaging that allow for layered z-stacked images of cells *in vitro* and methods we have developed to image *ex vivo* tumors provide an enhanced approach that allows for more advanced analysis of nanotubes connecting malignant cells in the stroma-rich tumor microenvironment (Figures 6B,C; Also see Movie S5). These visually detailed 3D images of mesothelioma cells provide further impetus for studying TnTs in this manner *in vitro*.

### Hyaluronic acid is not associated with TnTs

**Figure 7:**
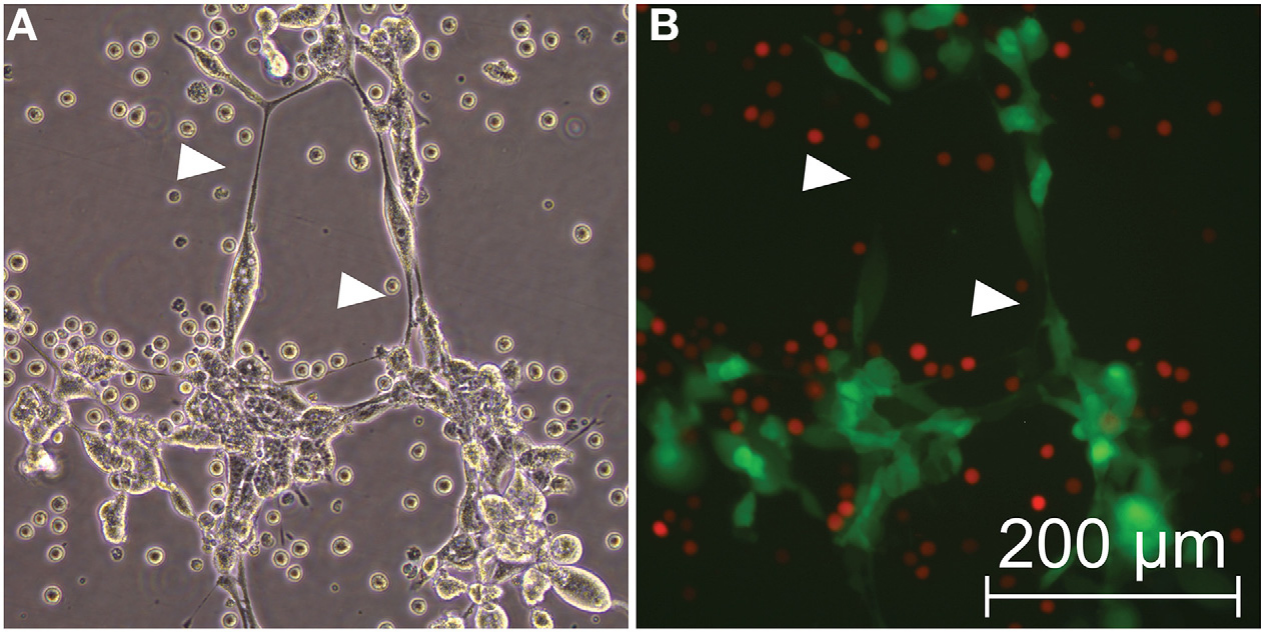
**Hyaluronic acid is not associated with TnTs**. U937 mononuclear cells (expressing Tomato Red) were added to cell culture growing MSTO-211H cells (expressing GFP) with TnTs. **(A)** Brightfield and **(B)** Fluorescent views of the same field of view are shown. Arrowheads indicate nanotubes.

Hyaluronic acid (HA), or hyaluronan, is a well-studied glycosaminoglycan that is secreted into the extracellular matrix; increased production of HA induces increased cell motility and an invasive phenotype in mesothelioma (Li and Heldin, 2001). Hyaluronan receptors are expressed preferentially on malignant mesothelioma cells but not on normal mesothelium (Asplund and Heldin, 1994). However, normal mesothelial cells and malignant cells derived from several organ sites synthesize relatively high quantities of hyaluronan, whose pericellular coat comprises bunches of short, adherent membranous protrusions consistent with actin-based stress fibers and microvilli (Kultti et al., 2006; Rilla et al., 2008). These coats create zones that have been well-described and are readily visualized microscopically using erythrocyte exclusion assays or the equivalent (McBride and Bard, 1979; Rilla et al., 2008). Conditions of cellular stress induced by either hyperglycemia, viral mimetic agent poly I:C, tunicamycin, or dextran sulfate, to name just several examples, have been shown to induce increased hyaluronan production and hyaluronan-based cellular “cables” that induce monocyte adhesion *in vitro* (Kultti et al., 2006); tunicamycin and dextran sulfate in particular induce endoplasmic reticulum-related metabolic stress that leads to increased production of hyaluronic acid, which in turn attracts and leads to increased adhesion of leukocytes via surface binding of CD44 (de La Motte et al., 1999; Majors et al., 2003; Wang and Hascall, 2004; Lauer et al., 2009). In our earliest studies examining what we later confirmed to be TnTs in mesothelioma, we performed standard exclusion assays using primary red blood cells (erythrocytes) or U937 lymphocyte (mononuclear) cells (Kultti et al., 2006; Rilla et al., 2008), but found no visual evidence of either pericellular zones or monocyte adhesion to TnT structures, indicating that TnTs were unlikely to harbor a significant amount of hyaluronan externally, also demonstrating that these entities are distinct from hyaluronan cables (Figure 7; Supplemental Figure S3). For fluorescent imaging and confirmation, we used MSTO-211H cells transfected with a lentivirus expressing GFP which we have used and described previously (Lou et al., 2012b) along with U937 cells transfected with a lentivirus expressing Tomato Red. To convincingly confirm that the extensions connecting cells were indeed TnTs, we performed time-lapse imaging that visibly demonstrated intercellular transfer of GFP (Movies S3, S4).

We also applied the HA-stimulating drug tunicamycin (final concentration 5 μg/ml) to MSTO cells in culture and examined these cells every 24 h up to 96 h. However, this led to no changes in TnT formation or morphology. Likewise, separate addition of dextran sulfate (10 μg/ml) to MSTO cells in culture led to cellular aggregation not unlike aggregates seen in patient effusion samples (Supplemental Figure S4). Poly I:C (10 μg/ml) induced a transformation of MSTO cells into a more mesenchymal, spindle-cell morphologic appearance without alteration of TnTs. This finding is consistent with similar effects of this drug on stimulating EMT in other cell types (Harada et al., 2009). We further treated MSTO cells with 4-methylumbelliferone (1.0 mM), an inhibitor of hyaluronan synthase and thus of hyaluronan cables (HAS) (Morohashi et al., 2006; Kultti et al., 2009); in separate wells we also assessed potential effects of the enzyme hyaluronidase (13 μM); neither drug had any effect on TnTs, consistent with the above data indicating HA does not play a notable role in TnT formation or maintenance (Supplemental Figure S4).

### Decreased tumor growth in mice implanted with TnT-primed mesothelioma cells also corresponds with decreased survival

**Figure 8:**
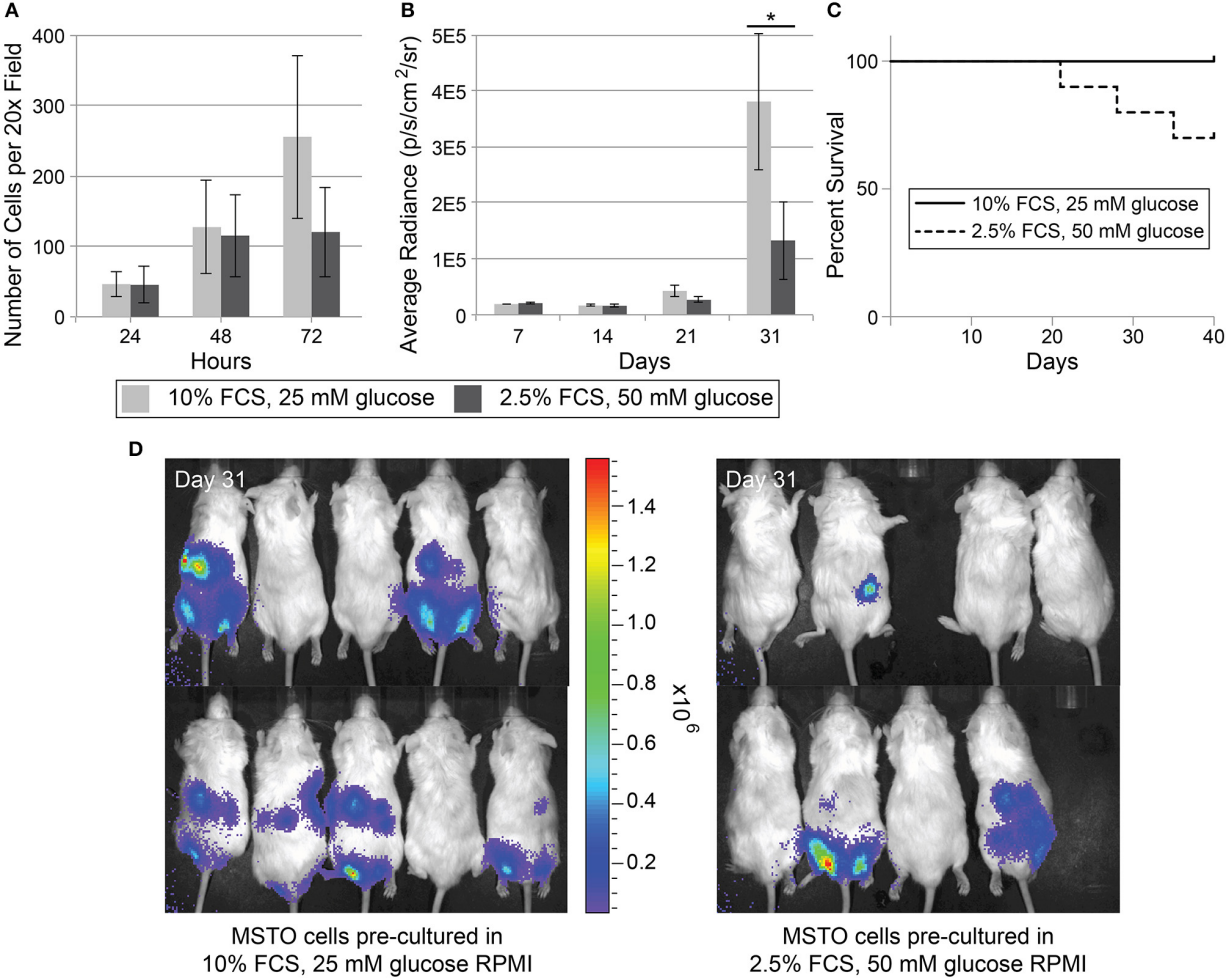
**Decreased tumor growth and survival of mice implanted with TnT-primed mesothelioma cells. (A)** *In vitro* cellular proliferation rate of MSTO-211H cells cultured in passage medium (10% FCS, 25 mM glucose RPMI) vs. TnT medium (2.5% FCS, 50mM glucose RPMI); **(B)** Average radiance of tumor growth at 7,14,21, and 31 days following peritoneal implantation in immunodeficient NOG mice of MSTO-211H cells cultured for 7 days using either passage medium or TnT medium; *indicates statistically significant difference between the two groups (*p* < 0.05); **(C)** Kaplan–Meier curve of survival of injected NOG mice, 10 mice in each group; **(D)** Bioimaging of NOG mice from each cohort.

Animal studies have identified nanotubes or similar structures *in vivo* in an inflammatory corneal mouse model (Chinnery et al., 2008; Seyed-Razavi et al., 2013) and *ex vivo* in adult mouse heart tissue (He et al., 2011), mouse alveoli (Islam et al., 2012), rabbit kidney parenchyma (Minuth and Denk, 2012), and mouse embryo non-neural ectoderm (Pyrgaki et al., 2010). Our group was the first to image TnTs in resected human tumor samples, initially on tumors from patients with MPM and lung adenocarcinoma (Lou et al., 2012b); we have been able to reproduce this finding using human mesothelioma tumors described in the current study (Movie S5). Our group has further extended demonstration of nanotube structures in malignant human ovarian tumors (Thayanithy et al., 2014a) as has another group (Pasquier et al., 2013). However, the technical difficulties of imaging nanotubes in the *in vivo* setting remain highly challenging. To assess effects of TnTs on *in vitro* cell proliferation, we used MSTO cells pre-conditioned in culture medium that we previously demonstrated increases the rate of TnT formation *in vitro* (Lou et al., 2012b). We conditioned MSTO cells in either low serum, hyperglycemic (2.5% FBS, 50 mM glucose) RPMI medium (referred to as “TnT medium”) or control passage RPMI medium (10% FBS, 25 mM glucose) for 7 days. This experiment demonstrated that proliferation of MSTO cells in low-serum, hyperglycemic medium was approximately half that of cells cultured in passage medium by 72 h (Figure 8A). To next examine the effect of TnTs on tumor growth *in vivo*, we used a NOG xenograft mouse model of malignant mesothelioma. We conditioned MSTO cells transfected with a lentivirus expressing luciferase in either TnT medium or control passage RPMI medium (10% FBS, 25 mM glucose) for 7 days. We then injected these cells into the peritoneal cavity of NOG immunocompromised mice. Mice were bioimaged every 7 days up to 31 days and the average radiance was measured; interestingly, by 31 days the mice injected with cells pre-conditioned in low-serum, hyperglycemic medium had developed less tumor burden than mice injected with the same cell line pre-conditioned in passage medium (Figure 8B). Thus, this *in vivo* finding mirrored the *in vitro* studies that demonstrated that proliferation of MSTO cells in low-serum, hyperglycemic medium was approximately half that of cells cultured in passage medium. Moreover, none of the NOG mice injected with control medium (*n* = 10) had died by day 31, but 2 mice injected with cells pre-conditioned in low serum, hyperglycemic medium (*n* = 10) had died by day 31 and an additional one died just after imaging (*p* = 0.067, Figure 8C); in a separate experiment repeating this approach, 5 of 10 mice with injected cells pre-conditioned in TnT medium died by Day 31, whereas 0 of 10 died by that day (data not shown). Using weight as a surrogate measure for morbidity, mice injected with cells primed with TnT medium displayed a sharper drop in weight over time than did mice injected with cells cultured in passaged medium (data not shown). Bioimages demonstrating the visual differences between the two groups are shown (Figure 8D). These *in vivo* findings set the stage for further evaluation of the potential role for TnTs in solid tumor malignancies, possibly by increasing the locoregional but not distant invasive capability of mesothelioma cells, with a mechanism independent of cell proliferation.

### Gene expression profiling of TnT-primed mesothelioma cells

**Figure 9:**
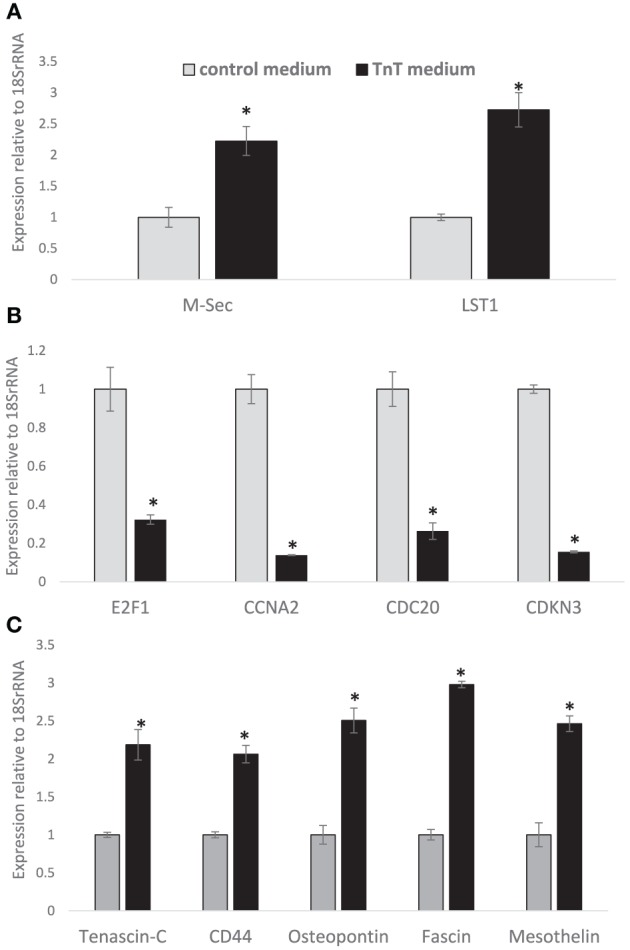
**Gene expression profiling of mesothelioma cells cultured in passage medium vs. TnT medium**. Expression of selected genes in MSTO cells grown in TnT media; samples were run in triplicate and means ± standard deviations are presented. Gray columns = results from cells grown in Control medium; black columns = results from cells grown in TnT medium. Expression of genes specific to TnTs **(A)**, cell cycle regulation **(B)**, and genes attributed to invasion, migration and metastasis **(C)** relative to cells grown in control media are shown. Note the significant downregulation of genes that positively modulate cell cycle in cells grown in TnT media. Conversely, genes attributed to cellular invasion, migration and metastasis functions are upregulated (^*^indicate *p*-values <0.05).

Due to the above findings, we next sought to determine relative differences in gene expression between MSTO cells conditioned in low serum, hyperglycemic (2.5 % FBS, 50 mM glucose) RPMI medium (i.e., “TnT medium”) or control passage RPMI medium (10% FBS, 25 mM glucose). We first investigated RNA levels of M-Sec (which is also called TNFaip2, or tumor necrosis factor-α-induced protein 2) and leukocyte specific transcript 1 (LST1), two gene products that are known to be enriched in TnTs (Hase et al., 2009; Schiller et al., 2013). Both genes were significantly upregulated in MSTO cells cultured in TnT medium compared to control medium (Figure 9A). We then investigated whether TnT medium, which is significantly lower in essential nutrients and also includes low percent of added serum (2.5% FBS) relative to passage medium (10% FBS), affects genes that promote cell cycle progression (Bracken et al., 2004; Nalepa et al., 2013). RNA levels of E2F1, its downstream targets CCNA2 and CDC20, and CDKN3 were significantly lower in MSTO cells grown in TnT medium than in cells grown in control medium (Figure 9B). This finding is consistent with our observation that cells grown in TnT medium undergo a lower rate of cell division. We next studied whether key genes involved in cellular migration, invasion, and metastasis are altered in mesothelioma cells cultured in TnT medium (Scholler et al., 1999; Rittling and Chambers, 2004; Guttery et al., 2010; Al-Alwan et al., 2011; Servais et al., 2012; Pietras et al., 2014). Relative to the MTSO cells grown in normal medium, tenascin-C, CD44, osteopontin, fascin, and mesothelin were all significantly induced in MSTO cells grown in TnT medium (Figure 9C). Further studies will evaluate whether induction of these genes in cells grown in TnT medium induce an adaptive gene expression profile leading to TnT formation and a higher propensity to invade, migrate, and metastasize.

## Discussion

Intercellular communication between cancer cells is crucial to the progression of invasive cancers, but the mechanisms by which communication occurs between distant and proximal cells in a tumor matrix remains poorly understood. TnTs are a novel candidate to explain how this communication process occurs (Lou et al., 2012a). Our prior and current work have consistently demonstrated TnTs in malignant mesothelioma and lung adenocarcinoma tumors from human patients (Lou et al., 2012b). This observation is consistent with the finding of another group that successfully imaged membrane nanotubes *in vivo* using an inflammatory cornea animal model (Chinnery et al., 2008). We have further demonstrated that TnTs are not exclusive to malignant mesothelioma and lung adenocarcinoma, but can form between malignant cells from a wide variety of histologic cancers, including pancreatic cancer as one example (Lou et al., 2013). In the present study, we describe our approaches to studying the relevance of TnTs in invasive malignancies, specifically in the context of MPM.

### TnT length as a function of the need for intercommunication among mesothelioma cells

When interpreting our findings of both cell proliferation rate and changes in TnT lengths, we take into account likely differences in natural biology and aggressiveness of the variable cell histologies (i.e., VAMT = sarcomatoid; H2052 = epithelioid, likely less aggressive; MSTO-211H = biphasic, encompassing features of both of VAMT and H2052). It is logical that as cells proliferate and are also motile, with less distance between cells over time, the average TnT length would decrease over time. The most aggressive cell line (VAMT, sarcomatoid) displayed the longest TnT length at 24 h (Figure 2), but not the highest proliferation rate by 72 h (Figure 1C); conversely, MSTO cells had the highest proliferation rate, but relatively shorter TnTs. Logically, this presents an interesting supposition: That cells proliferating at a low rate can be just as—if not more—aggressive, perhaps by forming longer TnTs and/or more TnTs. Knowing the clinical outcomes are worse with sarcomatoid variants than with other forms of mesothelioma, we postulate that this may hold true in the clinical setting. Further investigation is warranted based on these findings.

A key clinical manifestation of advanced thoracic malignancy (i.e., MPM and lung adenocarcinoma) is accumulation of pleural fluid that marks development of pleural effusions. Ascites is a similar process marked by accumulation of fluid in the abdominal cavity or peritoneum. In advanced cancers such as pleural or peritoneal mesothelioma, the accumulation of malignant fluid is a diagnostic and prognostic hallmark of aggressive disease. These effusions often contain a significant number of free-floating or suspended malignant cells; diagnosis can be made following cytologic examination of extracted fluid. However, beyond examination as a mere diagnostic marker, little is known about the role this specific cell population plays in the propagation of mesothelioma and disease progression. This population of free-floating, suspended cells is capable of undergoing epithelial-to-mesenchymal transition (EMT), thus increasing their invasive capability. In our prior study, we demonstrated that EMT effectively stimulates TnT formation in mesothelioma, as does acidic pH (Lou et al., 2012b) in the context of low-serum, hyperglycemic medium. This is especially important as low pH of pleural fluid derived from malignant effusions, including those derived from malignant mesothelioma, is a poor prognostic factor associated with decreased overall patient survival (Sahn and Good, 1988; Gottehrer et al., 1991).

In the present study, we examined extra-cytoplasmic actin extension of TnTs. We found that as mesothelioma cells proliferated the average TnT length decreased over time, possibly as a result of the low cellular requirement for long-distance connections among confluent cultures. Further, our *in vitro* model of pleural/peritoneal effusions indicated that TnTs may function as tethers that link suspended, non-adherent malignant cells to other suspended cells or adherent cells of the pleural lining of the thoracic cavity. These findings indicate that non-adherent viable cells in culture behave similarly to suspended effusion cells from mesothelioma patients and that these cells are capable of forming TnTs. Relative differences in TnT length may be a function of chemotactic factors promoting their formation and guiding their extension; a precedent has been established for this in correlation of length of cytonemes (actin-based filopodial protrusions similar to TnTs) to length of chemotactic gradients of Hedgehog signals in Drosophila wing disc models (Bischoff et al., 2013).

### Developing *in vitro* models that simulate potential TnT activity in an *in vivo* tumor microenvironment

To study the formation and function of TnTs, 3-dimensional *in vitro* models are needed to simulate the complex tumor microenvironment. A major challenge to 3-dimensional models of TnT signaling is the presence of other forms of external signaling that may confound study results. Exosomes, microvesicles, and other freely diffusible signals play an established role in intercellular signaling. Indeed, data recently published by our group demonstrated that tumor-derived exosomes stimulate TnT formation in mesothelioma cell culture (Thayanithy et al., 2014b). A previous report showed that other forms of external signaling could be excluded by culturing normal rat kidney cells in a viscous agarose matrix; this approach significantly decreased diffusion of extracellular signals, including from microvesicles or other free particles, while permitting TnT formation (Gurke et al., 2008). For the first time, in the current study we apply use of “TnT medium” (low-serum, hyperglycemic culture medium) to agarose in a relatively viscous microenvironment that not only remains permissive for, but also further induces, TnT formation in a manner that is reliable for further study. We propose using this approach for future studies that aim to minimize potential effect of exosomes and other diffusible factors that act as stimulatory signals and intercellular carriers of cargo, thus isolating examination of TnTs for specific analyses.

### TnTs as a therapeutic target for mesothelioma and other TnT-forming malignancies

TnTs are not exclusive to cancer and are a cellular entity in “normal,” non-malignant cells. For the purposes of studying the potential role of TnTs in cancer, we developed a tool to quantitatively assess TnT numbers that could be used as a “nanotube index” to study how increased “intercellular trafficking” of cellular cargo via TnTs is related to cell transformation and tumor formation. We adapted confocal microscopic techniques to visualize narrow nanotube structures. Quantitative assessment of TnTs/cell indicated that TnTs formed at a markedly higher rate among malignant mesothelioma cell lines than among normal mesothelial cells and in inverse proportion to the rate of cell proliferation. In fact, we found that the culture condition that most increased TnT numbers (low-serum, hyperglycemic medium, which we call “TnT medium”) did not lead to a corresponding increase in cell number, but rather a notable decrease in cell proliferation by approximately half. We attribute this decreased cell proliferation to both elements, i.e., low serum concentration (2.5% FBS) as well as high glucose, which induces reductions in cell proliferation as well as increased cell apoptosis of pericytes *in vitro* (Beltramo et al., 2006). These data indicate that TnT formation and cell proliferation are distinct processes that may occur during specific stages of malignant growth.

We previously used several inhibitors of pathways that have been implicated in actin-based cell invasion, including latrunculin A, an actin-destabilizing agent that is commonly used in *in vitro* studies and that has been used in multiple TnT studies (Tavi et al., 2010; Lou et al., 2012b; Vallabhaneni et al., 2012). We previously investigated potential metabolic pathways essential for TnT formation in mesothelioma and effectively demonstrated suppression of TnT formation using drugs that are used in the clinical setting for other malignancies, such as an mTOR inhibitor and the widely available drug metformin (Lou et al., 2012b), which stimulates AMP-activated protein kinase (AMPK) and thus indirectly stimulates the mTOR pathway as well (Zhou et al., 2001). This is particularly important in context of our findings in the current study that effusion-derived mesothelioma cells (from both cell lines and from human patients) form aggregate spheroids that are tethered by TnTs. *In vitro* 3-D spheroid models of mesothelioma demonstrate increased resistance to drugs and apoptosis compared to 2-dimensional cultures, and this is at least in part mediated by mTOR; however, inhibitors of mTOR can overcome this acquired resistance (Barbone et al., 2008; Wilson et al., 2008). Considering susceptibility of TnTs to mTOR inhibition, TnTs may play an important role in mediating chemoresistance of spheroids.

In the current study, we demonstrate that an additional rational drug—migrastatin—suppresses TnT formation in mesothelioma. Migrastatin has been found to be potent in blocking migration and metastasis of lung cancer in separate studies (Lecomte et al., 2011), providing impetus for further exploration of this drug as a potential novel therapeutic agent for both lung adenocarcinoma and MPM.

### Accurate *in vivo* assessment of TnT functions

*In vivo* examination of TnTs remains a major barrier to the study of the function of TnTs in cancer. *In vivo* studies of nanotubes have been limited to an inflammatory corneal model in mice (Chinnery et al., 2008; Seyed-Razavi et al., 2013), but successful intravital microscopy has been performed visualizing similar cellular extensions called cytonemes in Drosophila (Bischoff et al., 2013). We also developed specific protocols for imaging putative TnTs in human tumors, as our group's main research focus is cancer cell biology of highly invasive malignancies. In previous studies, we obtained 6 fresh intact tumor specimens from patients with either MPM or lung adenocarcinoma immediately following surgical resection. Using a Vibratome, we processed the specimens into thin cuts that were amenable to staining with Hoechst dye to visualize nuclei and MitoTracker Red to visualize the cell body and TnTs, which we had already demonstrated to work *in vitro* (Lou et al., 2012b). Of the six human patient-derived tumors, we detected TnTs in all six using confocal microscopy and 3-dimensional reconstruction using the Imaris software. TnTs have also been reported in ovarian tumor explants by our group and others (Pasquier et al., 2013; Thayanithy et al., 2014a). In the present study, cells primed in TnT medium had a lower proliferation rate *in vitro*; proliferation rate of these cells was similar when injected *in vivo*, as measured by lower detectable radiance. This is also supported by our finding that the level of genes involved in cell proliferation were also expressed at lower levels in cells that were primed in TnT medium compared to the cells primed with normal media (Figure 9). Although mice implanted with TnT-primed mesothelioma cells had lower overall disease burden, they also had poorer survival compared to mice implanted with mesothelioma cells pre-conditioned in control medium. Thus we postulate that a higher rate of formation of TnTs *in vivo* is associated with a higher level of local invasion of tumors, leading to lower survival in this animal model. Future work in our lab will follow up on these findings.This *in vivo* model will prove to be especially important to examining TnTs as a potential therapeutic target for treatment of cancer and subsequent studies for drug delivery via TnTs.

## Conclusions

In Conclusion, the mechanisms by which cells communicate with one another in the tumor microenvironment are not well understood (Bissell and Radisky, 2001; Pietras and Ostman, 2010; Bissell and Hines, 2011). Our works challenges the current paradigm that gap junctions, exosomes, or cytokines and other diffusible chemical signals are exclusive modes by which cells mediate intercellular communication in mesothelioma. Tunneling nanotubes are a novel biologic conduit for intercellular signaling and transport of cellular cargo. At this time, there appear to be more questions than answers in terms of what the mechanisms, functions, and role of these nano-sized structures are in various cell types. TnTs have attracted the interest of researchers across a spectrum of fields including neuroscience, immunology, infectious diseases, and cancer as described here. What remains unknown is how these diseases use TnTs to coordinate “social networking” among connected cells, which characteristics of TnTs are universal across cell types (e.g., cancer vs. non-cancer cells), and which aspects may be unique to the cell type studied. Researchers with interest in cellular communication may adapt their approach to the study of TnTs according to their research objectives. In this paper, we describe our approach to studying the relevance of TnTs in cancer, specifically in the context of the solid tumor matrix of aggressive malignancies.

### Conflict of interest statement

The authors declare that the research was conducted in the absence of any commercial or financial relationships that could be construed as a potential conflict of interest.

## Supplementary material

Movie S1 **Time-lapse microscopy imaging of intercellular transfer of mitochondria between mesothelioma cells connected via a TnT**. We used MitoTracker Red to stain MSTO-211H cells which were then cultured in hyperglycemic, low-serum (“TnT”) medium. Cells were allowed to adhere and form TnTs, which were then identified for analysis. Images were taken every 20 min for 24 h using a Perkin–Elmer spinning-disk confocal microscope.

Movie S2 **3-dimensional modeling of a nanotube**. Cells were stained with vimentin (red) and nuclear staining with Hoechst 33342 dye (blue) as described. Images were were taken as a z-stack at 40× and compiled using Imaris Viewer software.

Movie S3 **Intercellular transfer of GFP between mesothelioma cells connected via TnTs**. GFP-expressing (green) MSTO-211H cells were co-cultured with MSTO cells stained with the lipophilic dye DiI (red) as we have reported previously (Lou et al., 2012b) and cultured in 37°C, 5% CO_2_ for time-lapse imaging. Images were taken every 15 min for 5 h. In this sequence, the middle cell (green) is connected to two cells simultaneously via TnTs, which facilitate transfer of GFP both to and from that cell.

Movie S4 **Intercellular transfer of GFP via TnT connecting two MSTO-211H cells**. Higher-magnification view and time-lapse microscopy demonstrating bidirectional transfer of GFP between connected cells.

Movie S5 **3-dimensional reconstruction of a tumor surgically resected from a human patient with malignant pleural mesothelioma**. 3-dimensional imaging was performed using the Imaris Viewer.

## Abbreviations

MPM: malignant pleural mesothelioma
TnTs: tunneling nanotubes
nm: nanometers
EMT: epithelial-to-mesenchymal transition
IF: immunofluorescence
FBS: fetal bovine serum
HA: hyaluronic acid
GFP: green fluorescent protein
EM: electron microscopy
TNFaip2: tumor necrosis factor-alpha-induced protein 2 (also called M-Sec), MSKCC, Memorial Sloan-Kettering Cancer Center.
