# Supplementary material for: Intercellular communication in malignant pleural mesothelioma: properties of tunneling nanotubes
Source: Front Physiol. 2014 Oct 31;5:400. doi: 10.3389/fphys.2014.00400 (PMC4215694; doi:10.3389/fphys.2014.00400)
Supplement: Supplementary file 6 [file DataSheet1.DOCX]

**Supplementary Table 1. Nucleotide sequence of primers used for q-RT PCR**

| **No** | **Name** | **Sequence (5’ to 3’ direction)** |
| --- | --- | --- |
|  | 18srRNA-F | TCAAGAACGAAAGTCGGAGG |
|  | 18srRNA-R | GGACATCTAAGGGCATCACA |
|  | LST1-F | TGCCTGTGTTGGCTGCATCGAA |
|  | LST1-R | GAGGTCAGGTCCCTCACTGCT |
|  | M-Sec-F | CCTGCTCTCCCTACGC |
|  | M-Sec-R | CGTCCAAGATGCTCCG |
|  | E2F1-F | ACGTGACGTGTCAGGACCT |
|  | E2F1-R | GATCGGGCCTTGTTTGCTCTT |
|  | CCNA2-F | CGCTGGCGGTACTGAAGTC |
|  | CCNA2-R | GAGGAACGGTGACATGCTCAT |
|  | CDC20-F | GACCACTCCTAGCAAACCTGG |
|  | CDC20-R | GGGCGTCTGGCTGTTTTCA |
|  | CDKN3-F | CCAGCAATGTGGAATTATCACCC |
|  | CDKN3-R | GCAGCTAATTTGTCCCGAAACTC |
|  | Tenascin-C-F | TGGTGGAGAACACTGGCTATGAC |
|  | Tenascin-C-R | GGGATCCCCAGCCAAGGT |
|  | CD44-F | AGAAGGTGTGGGCAGAAGAA |
|  | CD44-R | AAATGCACCATTTCCTGAGA |
|  | Osteopontin-F | TTGCAGCCTTCTCAGCCAA |
|  | Osteopontin-R | GGAGGCAAAAGCAAATCACTG |
|  | Fascin-F | ATGTTGCCCAGGTTGAACTC |
|  | Fascin-R | TCACACCTGAAATCCCAACA |
|  | Mesothelin-F | AACGGCTACCTGGTCCTAG |
|  | Mesothelin-R | TTTACTGAGCGCGAGTTCTC |
