# Supplementary figures and images for: Intercellular communication in malignant pleural mesothelioma: properties of tunneling nanotubes
Source: Front Physiol. 2014 Oct 31;5:400. doi: 10.3389/fphys.2014.00400 (PMC4215694; doi:10.3389/fphys.2014.00400)

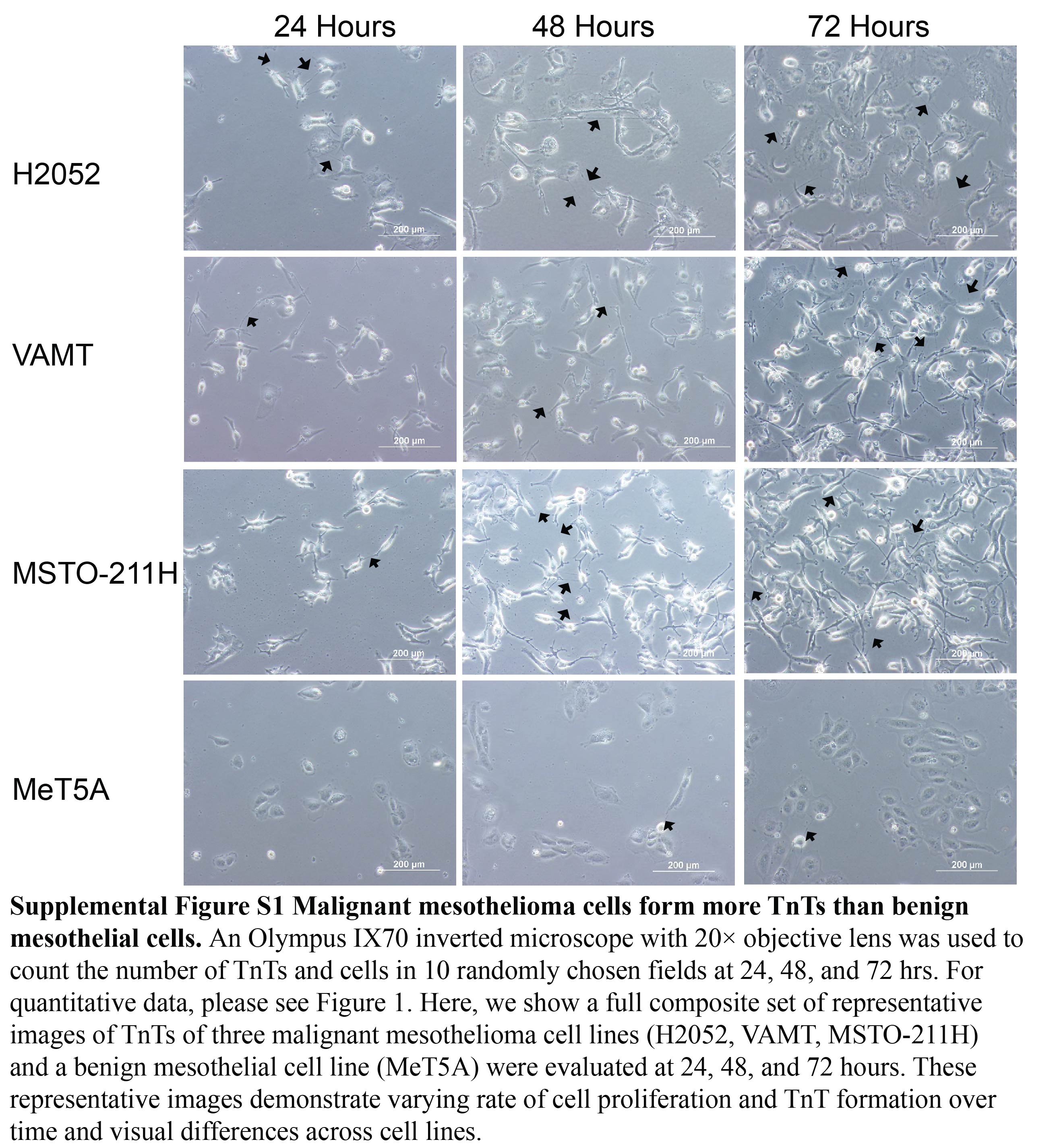

Supplement: Supplementary file 7 [file Image1.JPEG]

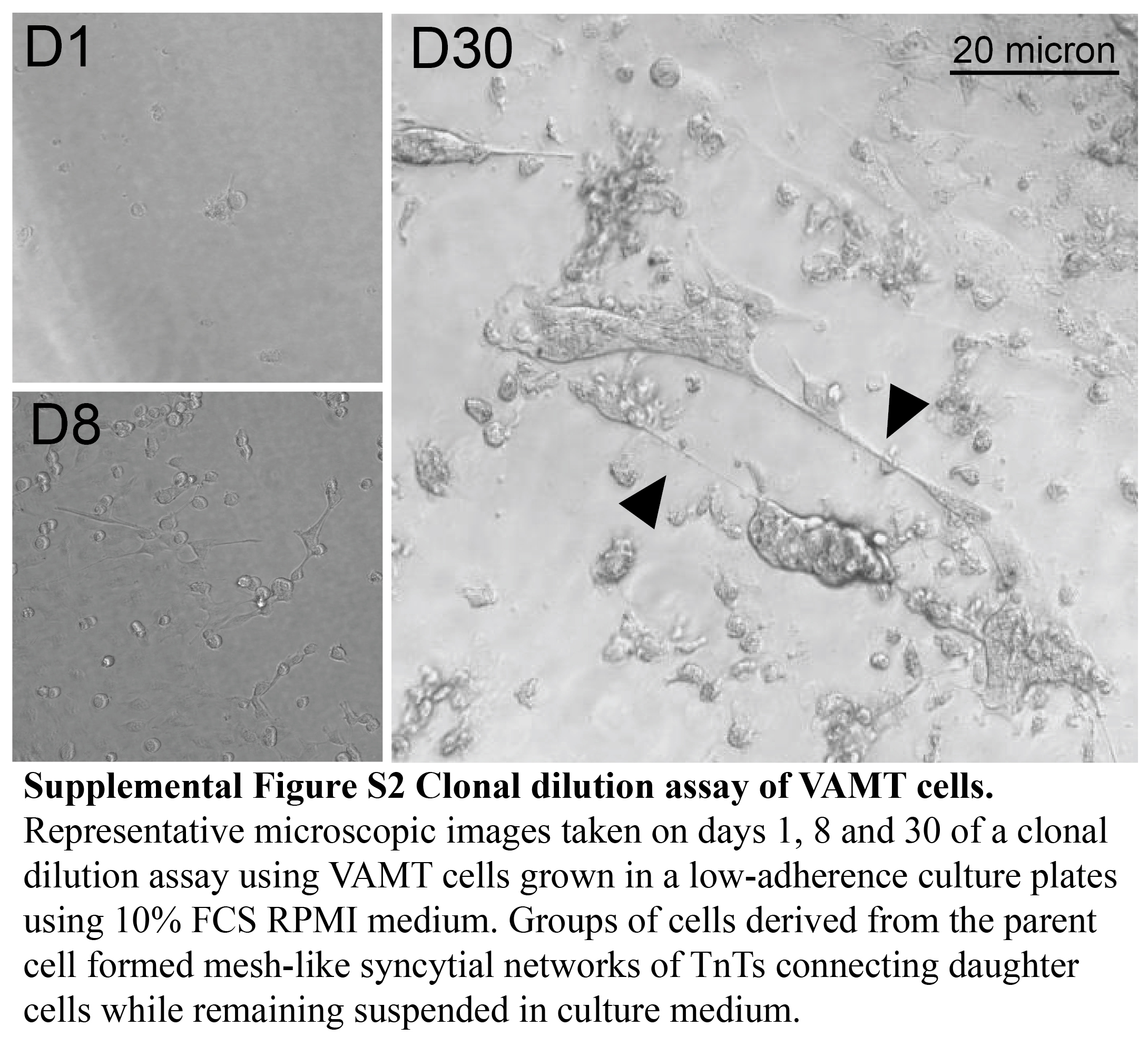

Supplement: Supplementary file 8 [file Image2.JPEG]

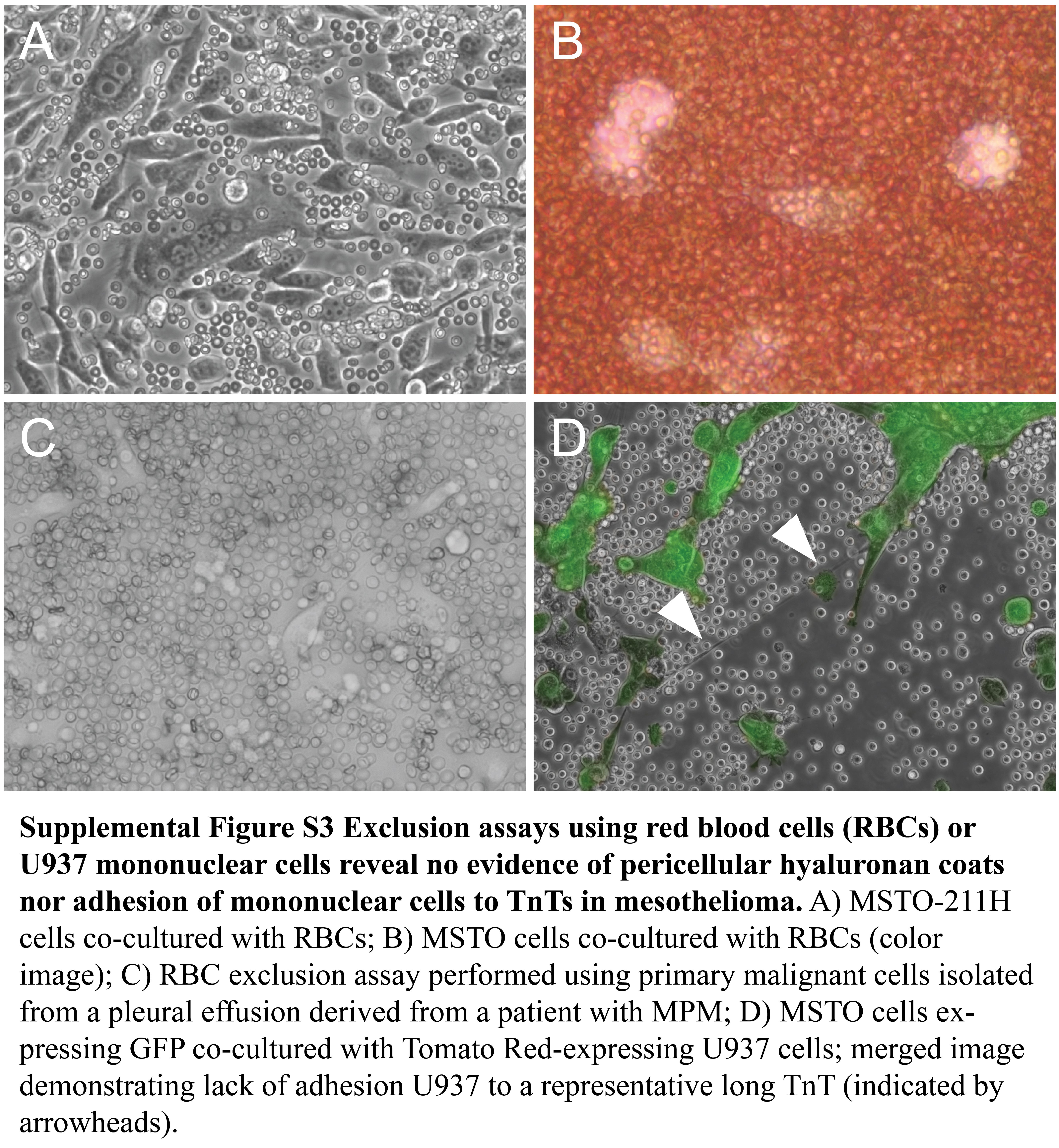

Supplement: Supplementary file 9 [file Image3.JPEG]

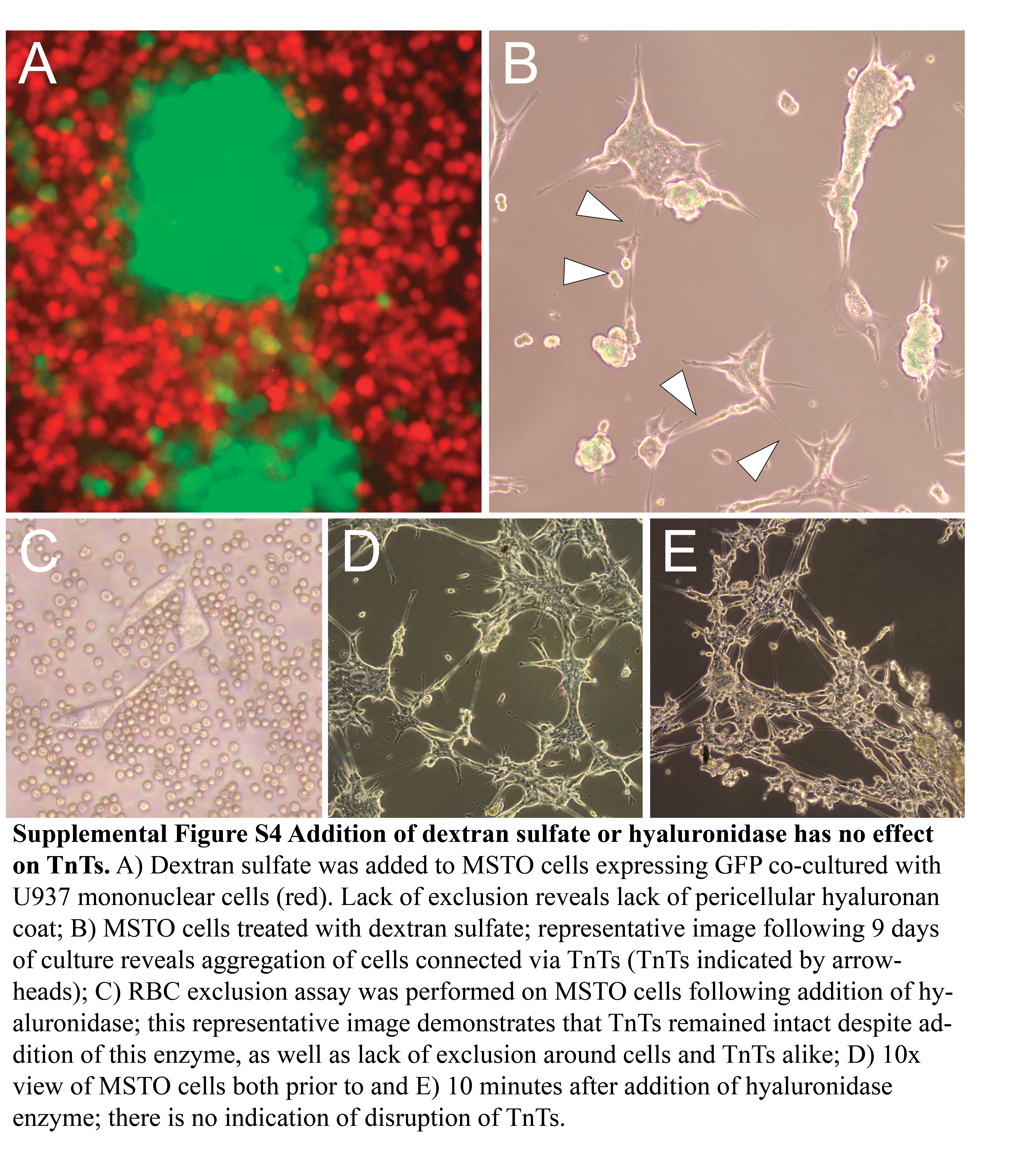

Supplement: Supplementary file 10 [file Image4.JPEG]
